# Tranexamic Acid in the Treatment of Hip Fractures: A Clinical Review

**DOI:** 10.51894/001c.7026

**Published:** 2019-03-04

**Authors:** Nathan M. Krebs, Michael J. VanWagner, Tiffany Marchewka, Usama Faraj, Christopher R. Vitale

**Affiliations:** 1 McLaren Macomb Orthopedic Surgery Resident, Michigan State University Statewide Campus System; 2 Michigan State University College of Osteopathic Medicine Medical Student; 3 McLaren Macomb Orthopedic Trauma Surgeon

**Keywords:** blood transfusion, hip fractures, tranexamic acid

## Abstract

**INTRODUCTION:**

Although regularly used as a method to reduce blood loss in elective orthopedic procedures (e.g., total hip and knee arthroplasty), there currently is little evidence concerning the optimal dosage, timing and route for the use of tranexamic acid to reduce postoperative blood loss in hip fracture repair.

**SUMMARY OF THE EVIDENCE:**

The current literature suggests that tranexamic acid may be used to reduce postoperative blood loss in addition to reducing the risk of requiring blood transfusions following the surgical repair of hip fractures. Furthermore, it may have the potential to improve patient outcomes and decrease the overall costs of caring for this patient population.

**CONCLUSIONS:**

Further studies are needed to truly gauge the effect of tranexamic acid on long-term patient outcomes and hospital costs.

## INTRODUCTION

There are approximately 300,000 patients annually hospitalized for hip fractures in the United States.^1^ As the vast majority of these injuries are treated surgically, this creates a significant burden on our healthcare system. Hip fractures are commonly associated with a relatively large amount of blood loss from the initial injury in addition to blood loss resulting from surgery.^2^ A range of between 20-60% of patients require blood transfusions after surgery, which may result in an increase in postoperative infections, increased length of hospital stay, and potential increases in admission costs by an average of approximately $1731.^2^ Serine proteases has been employed in an attempt to control postoperative hemorrhage, but with the risk of anaphylaxis, this option was not ideal.^3^ The 2010 CRASH-2 trial tested the use of tranexamic acid (TXA), a lysine analog that has been showing promise in many aspects of orthopedics.

TXA acts as an antifibrinolytic agent by competitively inhibiting the conversion of plasminogen to plasmin, acting to minimize postoperative bleeding.^4^ Although now more regularly used as a method to reduce blood loss in elective orthopedic procedures (e.g., total hip and knee arthroplasty), there is currently little evidence concerning the optimal use (i.e., dosage, timing and route) of TXA in hip fracture repair.^5^ TXA has been thought to be a viable strategy to minimize blood loss and decrease the subsequent need for blood transfusion postoperatively. Furthermore, TXA use may improve patient outcomes by avoiding the morbidity and mortality associated with blood transfusions (e.g., allergic and hemolytic reactions, fever, etc.) and reduce the total costs of care for these patients.^1^

## SUMMARY OF THE EVIDENCE

The use of TXA in hip, knee, and shoulder arthroplasty as a way to decrease perioperative blood loss and reduce the need for postoperative blood transfusion has been well documented in the literature.^5–8^ There have also been studies demonstrating decreased blood loss and transfusion rates with TXA use in trauma and pelvic fractures.^8–10^ The current literature involving TXA use in the treatment of hip fractures is relatively sparse. Approximately 20% of all orthopedic trauma is hip fracture related.^11^ Postoperative blood loss can range from 300 to 1000 milliliters after undergoing surgical fixation secondary to over-activation of the fibrinolytic system from the initial injury in combination with the additional insult from the surgery.^12^

TXA has been less frequently used by orthopedic surgeons in trauma procedures compared with elective procedures because of the increased activation of the fibrinolytic system and subsequent risk of venothromboembolism (VTE).^1^ VTE is defined as a condition when a blood clot forms in the venous system, typically the legs, then becomes dislodged and travels to the lungs. However, data continues to amass in support of using TXA in patients with hip fractures.^1^

In 2017, Watts et al. examined the use of TXA in patients with acute femoral neck fractures treated with hemiarthroplasty or total hip arthroplasty.^13^ A hemiarthroplasty is a partial hip replacement that involves replacing the proximal end of the femur and leaves the native acetabulum intact. This is in contrast to a total hip arthroplasty, which involves replacing the proximal femur in addition to placing a prosthetic cup into the acetabulum.

**Table:** Figure 1 Box Summary

Clinical Question:
What role does tranexamic acid have in the treatment of hip fractures?
**Current Evidence:**
The current literature suggests that tranexamic acid may be used to reduce postoperative blood loss in addition to reducing the risk of requiring blood transfusions following the surgical treatment of hip fractures. Furthermore, it may have the potential to improve patient outcomes and decrease the overall costs of caring for this patient population.
**Take Home Message:**
The current literature supports the use of tranexamic acid in the treatment of hip fractures in patients without the known contraindications. Further studies are needed to truly define its effect on long-term outcomes and hospital costs.

**Table:** Table 1 Recent Studies on TXA Use in the Surgical Treatment of Hip Fractures

**Study**	**Design**	**Hip Fracture**	**Blood Loss**	**Blood Transfusion**	**Adverse Events**
*Watts et al. 2017*	RCT with 138 patients	Arthroplasty for femoral neck fracture	Reduced by 305 mL in TXA group (p=0.0005)	Reduced by 9% in TXA group (p=0.22)	No increased risk with TXA
*Drakos et al. 2017*	RCT with 200 patients	IT fracture treated with DHS or CMN	Reduced HCT loss by 2.5 in TXA group (p<0.01)	Reduced by 43% in TXA group (p<0.01)	No increased risk with TXA
*Virani et al. 2016*	RCT with 137 patients	IT fracture treated with CMN	No significant difference in blood loss	No significant difference in transfusion rate	No increased risk with TXA
*Schiavone et al. 2018*	RCT with 90 patients	IT fracture treated with CMN	n/a	Reduced by 18% in TXA group (p<0.05)	No increased risk with TXA
*Tengberg et al. 2016*	RCT with 72 patients	IT fracture treated with CMN	Reduced by 570 mL in TXA group (p=0.029)	Reduced by 0.6% in TXA group (p=0.21)	No increased risk with TXA
*Barauh et al. 2016*	RCT with 60 patients	IT fracture treated with DHS	Reduced by 270 mL in TXA group (p<0.001)	n/a	No increased risk with TXA
*Lee at al. 2015*	Retrospective cohort study of 271 patients	Hemiarthroplasty for femoral neck fractures	Reduced drop in hemoglobin (<2g/dl) in TXA group (p=0.014)	Reduced by 13% in TXA group (p=0.005)	No increased risk with TXA
*Mohib et al. 2015*	RCT with 100 patients	IT fractures treated with DHS or CMN	Reduced hemoglobin loss by 1.3 g/dl in TXA group (p=0.007)	Reduced by 24% in TXA group (p=0.009)	No increased risk with TXA

RCT: Randomized Controlled Trial

IT: Intertrochanteric

DHS: Dynamic Hip Screw

CMN: Cephalomedullary nail

TXA: Tranexamic Acid

n/a: not available

Statistical significance: p<0.05

These authors concluded that 15 milligram/kilogram intravenous (IV) TXA given at the time of incision and just before wound closure decreased postoperative transfusion rates when compared to the control group.^13^ In 2017, Wang et al. conducted another meta-analysis that found similar results in patients with intertrochanteric hip fractures treated with dynamic hip screw or cephalomedullary nail when given IV TXA.^14^ An intertrochanteric hip fracture is an extra-capsular fracture of the proximal femur that extends obliquely from the greater to lesser trochanter. A cephalomedullary nail is a device that consists of an intramedullary rod with a lag screw that goes through the nail in to the femoral head.

Drakos et al. also studied the efficacy of TXA in patients with intertrochanteric femur fractures treated with a cephalomedullary nail and noticed a 43% reduction in the number of transfusions in patients who received TXA.^15^ These patients received 3.0 grams of TXA injected under the deep fascia near the fracture site. For those patients who required blood transfusions, they received fewer units of blood compared to the control group. This study group also found the use of TXA to be cost effective, saving the hospital 77 euros (89.14 USD) per patient.^15^

Another concern with hip fractures is the phenomenon of “hidden blood loss.”^16^ The amount of hidden blood loss depends on the severity of the injury and the treatment, whether it be cannulated screws, dynamic hip screw, cephalomedullary nail, or arthroplasty.^16^ In 2018, Lei et. al. demonstrated that patients treated with a cephalomedullary nail who received 1.0 gram of IV TXA prior to incision had an average lower hidden blood loss of 210 milliliters compared to the control group, which had 359 milliliters. Additionally, they noted that the transfusion rates decreased approximately 50% in those who received TXA.^16^

This is in contrast to a 2016 study by Viriani et al., that found no statistically significant difference in average postoperative blood loss or hemoglobin levels when 2.0 grams of TXA was administered at the fracture site in patients treated with dynamic hip screw and barrel plating.^17^ This study remains one of the few in which TXA has not shown to be beneficial. This may be related to the conclusion that an increase in inflammatory markers, specifically the acute phase protein alpha-1 acid glycoprotein, is higher in this population of patients and may negate the use of TXA.^18^

In 2018, Schiavone et al. showed that patients with intertrochanteric femur fractures had a decrease in the percentage of hemoglobin lost postoperatively, although not a statistically significant difference in decreased transfusion rates when administered IV TXA.^19^ The group also noted that TXA use in their sample patients with intertrochanteric femur fractures experienced an increase in VTE, although these findings were not statistically significant.^19^ Tenberg et al. (2016) treated intertrochanteric femur fractures with cephalomedullary nails and administered 1.0 gram of IV TXA preoperatively and 3.0 grams of IV TXA postoperatively.^18^ These authors noted a mean blood loss reduction of 570 milliliters compared to 2100 milliliters in patients who had not received TXA. Of note, this study did show an increase in the 90-day mortality rate in the experimental group, although the increase was not quite statistically significant, p = 0.07.^18^

A 2016 study by Baruah et al. demonstrated decreased postoperative total blood loss in intertrochanteric hip fractures fixed with dynamic hip screws when given a single dose of 15 milligram/kilogram IV TXA fifteen minutes prior to surgery.^20^ The authors noted that there were no VTE events in either the control group or experimental group patients who received TXA.^20^ Similar results were found in another randomized controlled trial including 271 patients with femoral neck fractures managed with hemiarthroplasty.^21^ These patients were given a 1.0 gram preoperative IV bolus of TXA and found to have decreased total blood loss and decreased transfusion rates. Additionally, they identified no difference in 30 or 90-day mortality between the TXA and control groups.^21^

In 2015, Mohib et al. conducted an observational cohort study of 100 patients with hip fractures.^22^ These patients were given two IV dosages of 15 milligram/kilogram TXA, one before incision and one three hours after the initial dosage. The patients treated with TXA had a mean postoperative hemoglobin of 10.2 grams/deciliter compared to the mean of 8.9 grams/deciliter in the control group (p = 0.007). There was also a 42% reduction in the transfusion rate in those patients receiving TXA (p = 0.009).^22^

TXA is typically well tolerated in patients. However, the optimal dosing regimen has not yet been clearly defined in the literature, with regards to timing and route of administration.^1^ Absolute contraindications to TXA include allergy to TXA as well as a concurrent subarachnoid hemorrhage.^10^ The use of IV TXA in patients with a history of VTE or PE, ischemic TIA, acute MI, or known seizure disorder is relatively contraindicated.^23–25^

## CONCLUSIONS

Hip fractures continue to place a significant healthcare cost burden on our healthcare system. Much attention has been placed on the timing and type of surgical intervention in these patients, as well as an overall team approach to managing them. However, with rising life expectancy and an increased number of people living with chronic health conditions, the prevalence of hip fractures will continue to rise. The benefits of TXA in elective hip and knee arthroplasty, as well as the general trauma literature are well documented, and its use has become a standard at most institutions. Despite this, the use of TXA is not as widely utilized in the hip fracture population due to the concerns of possible increased risk of VTE.

This clinical review examined much of the current data involving TXA in the hip fracture population. The majority of the current literature supports the use of TXA in patients with hip fractures, with evidence generally suggesting decreased transfusion rates and blood loss in these patients. However, unlike elective hip and knee arthroplasty patients, hip fracture patients are a less controlled group. These are frequently not elective surgeries and as a result, they are often a higher risk population for perioperative morbidity and mortality.^1^

Furthermore, hip fracture patients experience a double-insult of blood loss, both from the initial fracture, as well as the surgical intervention.^2^ This makes it more difficult to evaluate the efficacy of TXA in these patients, as timing of TXA administration is paramount and well established in the general trauma literature. Further randomized controlled trials are needed to help define the optimal timing and route of TXA use in varied types of hip fracture patients. These studies could further investigate TXA administration in this population within an established window of the sentinel event injury, as well as perioperatively.

The current literature is encouraging, and generally supports the use of TXA in hip fracture patients without contraindications. As orthopedic surgeons will continue to strive to decrease the significant morbidity and mortality seen in the hip fracture population, TXA appears to be a safe and effective medication to decrease both postoperative blood loss and minimize blood transfusions after hip fracture surgery.

### Conflict of Interest

The authors declare no conflict of interest.
